# Wrist Bone Motion during Flexion-Extension and Radial-Ulnar Deviation: An MRI Study

**DOI:** 10.3390/life12101458

**Published:** 2022-09-20

**Authors:** Jianzhang Li, Björn Rath, Frank Hildebrand, Jörg Eschweiler

**Affiliations:** 1 Department of Orthopaedics, Trauma and Reconstructive Surgery, RWTH Aachen University Hospital, 52074 Aachen, Germany; 2 Department of Orthopaedic Surgery, Klinikum Wels-Grieskirchen, 4600 Wels, Austria

**Keywords:** wrist cartilage, helical axis, wrist kinematics, wrist model, motion analysis

## Abstract

The wrist joint plays a vital role in activities of daily living. Clinical applications, e.g., therapeutic planning, prosthesis design, and wrist biomechanical analysis, require a detailed understanding of wrist maneuvers and motion. The lack of soft tissue information, motion analysis on limited carpal bones, etc., restrain the investigation of wrist kinematics. In this study, we established 3D models of carpal bones with their cartilages, and revealed the helical axes (HA) of all eight carpal bones for the first time. Both left and right hands at different positions of flexion-extension (FE) and radial-ulnar deviation (RUD) from five subjects were *in-vivo* imaged through a magnetic resonance imaging device. We segmented all of the bones, including cartilage information in the wrist joint, after which we explored the kinematics of all carpal bones with the HA method. The results showed that the HA of all carpal bones for FE bounded tightly and was mainly located slightly above the radius. During the RUD, carpal bones in the distal row rotated along with wrist movement while the scaphoid, lunate, and triquetrum primarily flexed and extended. Further results reported that the carpal bones translated greater in RUD than in FE. With the generation of more delicate wrist models and thorough investigations of carpal motion, a better understanding of wrist kinematics was obtained for further pathologic assessment and surgical treatment.

## 1. Introduction

Consisting of eight small bones, the wrist is one of the most complex joints in the human musculoskeletal system. The wrist can be roughly categorized into a two degrees-of-freedom (DoF) joint, as its motions are mainly flexion and extension (FE) and radial and ulnar deviation (RUD). Normal wrist function is generated by the interplay between the carpal bones, the radius and ulnar, and their affiliated soft tissue. Therefore, hiding under the two DoFs, 48 DoFs of the eight carpal bones (six for each bone) remain an increasing research interest. For clinical practices, such as therapeutic planning [1], surgical assessment [2,3], and prosthesis design [4], the representation of the joint spatial information is highly essential, i.e., the anatomical structure and kinematics of carpal bones.

To obtain the carpal kinematics, attempts, including *in-vitro* invasive studies by inserting an electromagnetic tracking system [3] or Kirschner wires [5], had analyzed the motion upon limited carpal bones, e.g., only on capitate and scaphoid. Improvement in clinical diagnosis and treatment has been reported by introducing medical imaging techniques, e.g., computerized tomography (CT) and magnetic resonance imaging (MRI) [6]. Thus, non-invasive intervention plays a major role in the present day kinematical study of the wrist, including radiological imaging methods and optical tracking systems [7]. Former methods are mainly confined to the utilization of CT [8,9,10,11,12], resulting in the lack of soft tissue information. Meanwhile, to avoid radiation exposure, limited wrist positions were taken into consideration when using CT [12], which can only cover part of the wrist’s range of motion (RoM). The latter method can obtain the whole wrist RoM but merely the rough movement without real bone kinematics. Therefore, increasing investigations using MRI have been found recently [13,14], which covered the whole wrist RoM, however, excluding the effect of carpal cartilages. As simulated in our previous model [1], soft tissue undertook essential parts regarding model kinematical simulation performance. The absence of the soft tissue and the entire RoM weakens the understanding of wrist functionality.

A common depiction of wrist kinematics includes rigid body transformation (RBT) [8,15], rotation angle description [5,9], and helical axis (HA) [5,7]. Compared to the rotation angle description, the RBT offers a more comprehensive illustration of individual bone motion. With the transformation matrix, multiple motions in both forward- and inverse-kinematics can be mathematically obtained [16]. Such a method also has a good generalization ability, as it can be applied on any three-dimensional rigid body joint kinematic analysis [15]. Despite its unique representation, the RBT highly depends on the choice of the coordinate system and specific rotation sequences and, therefore, is less intuitive than the HA analysis [17]. The HA can be decomposed into a uniform rotation around an axis and a translation along the same axis [18]. It has been extensively utilized in medical practice, since its rotation and translation are invariant to coordinate transformations [17].

Although the HA has been analyzed in various studies [5,12,13,14,19], it was restricted to the limited carpal bones, e.g., the scaphoid, lunate, and capitate. Therefore, one purpose of this study is to reveal the HA on all eight carpal bones and establish a more delicate wrist model by filling the inter-bone gap with cartilage. Meanwhile, although left and right hands have been studied, the hypothesis that no difference exists between them has not been statistically studied.

Three main contributions are built: (1) we acquired *in-vivo* MR images on both the left and right wrists from five subjects at various wrist positions, covering the whole wrist RoM; (2) we segmented all the wrist bones with their cartilages with high accuracy, generating a wrist model with soft tissue for the first time; (3) we extensively investigated the HA of all eight carpal bones regarding all acquired motion for the first time and statistically confirmed the hypothesis that no motion difference existed between the left and right hand.

## 2. Materials and Methods

### 2.1. Subjects

This *in-vivo* study was approved by the authors’ institutional review board (EK 171/10) in 2017 and written informed consent requirements were obtained. In total, five healthy volunteers (four male, aging from 22 to 31; one female, age 24, mean age 26.6 with standard deviation 3.6) without any known prior wrist injury were recruited for the research. Both left and right wrists were imaged.

### 2.2. Wrist Positioning Protocol and Acquisition

**Figure 1:**
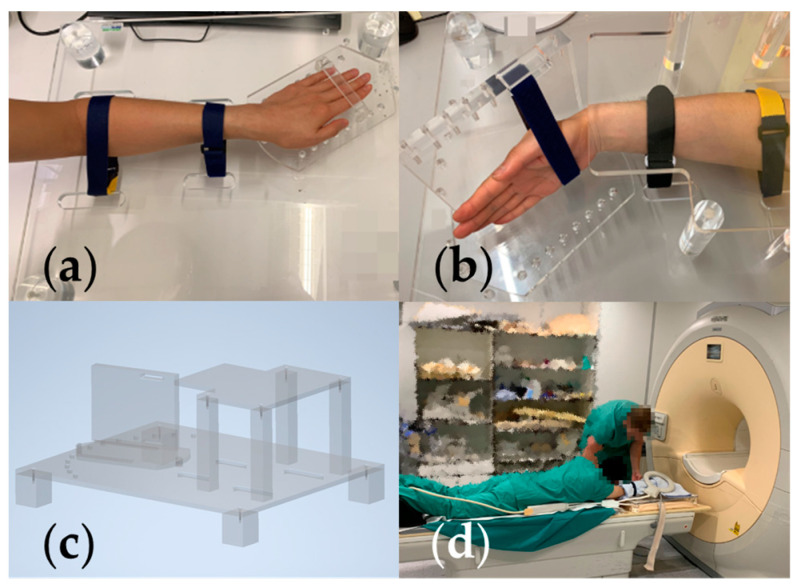
(**a**) Holding device set-up for RUD, the one demonstrated is an ulnar deviation (UD) 20°. (**b**) Holding device set-up for FE, the one demonstrated is flexion 30°. (**c**) 3D model of the holding device. (**d**) Participant posture during acquisition.

To entirely cover the RoM of the wrist joint and minimize effects on positioning caused by individuals, we designed a special holding device for wrist positioning (Figure 1c). The device mainly consisted of three parts: (1) a base that holds a rotation tray and fixes the forearm; (2) the rotating tray that can be adapted for either FE or RUD for both hands; the rotation angle resolution equals 10°; (3) a fixing pin that defines and retains the rotation angle. During the acquisition, the middle point of the radio-scaphoid fossa and radio-lunate fossa overlapped the rotation pivot of the rotation tray. All fingers are extended and adducted with the middle finger directed to the fixing pin. The participant lay in a prone position with the hand to be imaged outstretched ahead (“the superman pose”) (Figure 1d).

We defined the movement toward flexion and radial deviation as positive towards extension, and ulnar deviation as negative. The wrist positioning protocols included scans for FE at 0° (neutral wrist position), ±30°, and ±60°; for RUD at 0° (neutral wrist position), +10°, ±20°, and −40°.

All the *in-vivo* datasets were imaged using a high-resolution 3D-WATSc (water selective cartilage scans) sequence on a clinical 3-Tesla MRI scanner (Achieva, Philips Healthcare, Best, The Netherlands). These images were acquired with a slice thickness of 1.5 mm and a slice increment of 0.75 mm, a field of view of 180 mm × 180 mm, an acquisition matrix of 368 × 368 pixels, a flip angle of 17°, a repetition time of 9.9 ms, and an echo time of 5.1 ms. The voxel size is 0.35 mm × 0.35 mm × 0.75 mm.

### 2.3. Image Processing and Model Surface Refinement

**Figure 2:**
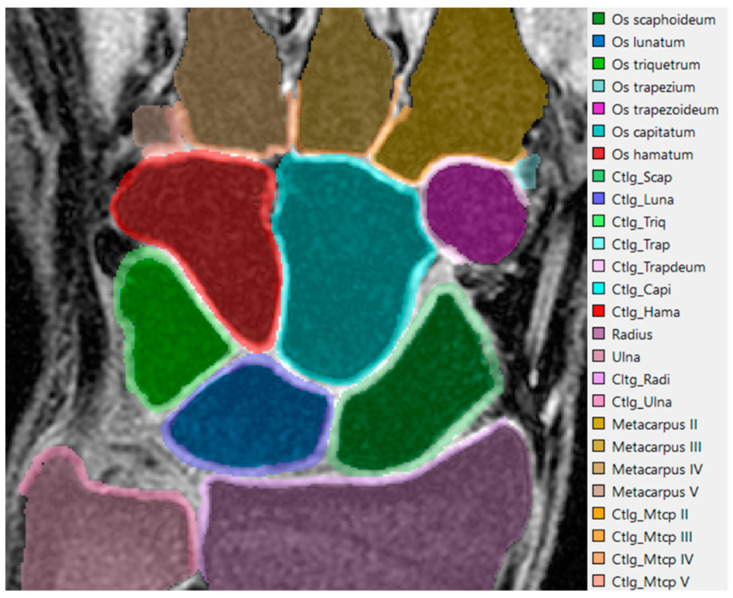
An exemplary segmented MR image slice of the wrist, the right side is the corresponding labels.

**Figure 3:**
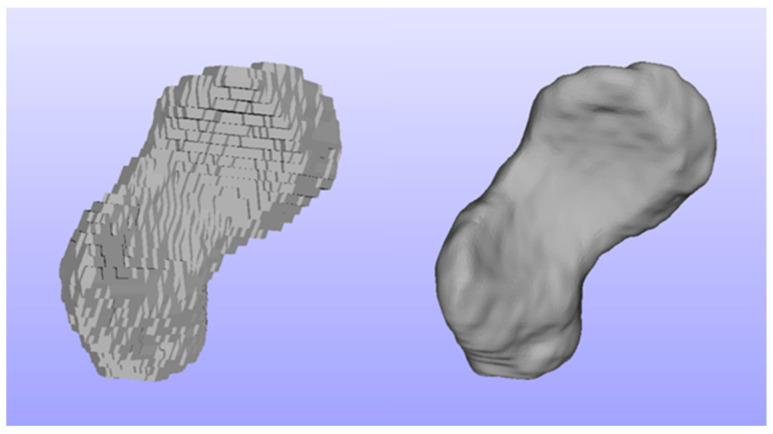
An exemplary scaphoid bone before (**left**) and after (**right**) smoothing.

We performed manual segmentation of the wrist bones and corresponding cartilages in the MR images (Figure 2). The manual segmentations were applied in the free, open-source platform ITK-SNAP (version 3.8) [20]. The unprocessed carpal and cartilage models were directly outputted from the ITK-SNAP without any further build-in smoothing procedure. Then, we utilized our former image processing strategy [21,22] for the model surface refinement (Figure 3), which offers the best compromise between model surface smoothness and boney surface fidelity.

### 2.4. Radius-Based Registration and Carpal Model Description

**Figure 4:**
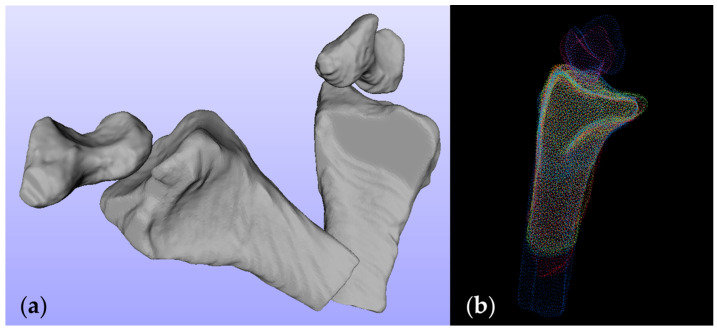
(**a**) Two radii and scaphoid bones from the same subject of different acquisition protocols. (**b**) Ten aligned radii and two scaphoid bones from four subjects of different acquisition protocols. Each color represents one bone.

The original position and orientation of the processed models can vary significantly due to the configuration of the built-in coordinate system in the MRI scanner, the individual movement of participants during acquisition, etc., (Figure 4a). Therefore, we converted all the radii to point clouds (minimal count of points for a radius model: 15943) and perform the coherent point drift (CPD) algorithm [23] to align all other radii to the randomly selected radius reference (Figure 4b). The CPD algorithm is a probabilistic method that considers the alignment as a probability density estimation problem. Briefly, given two pointsets to be registered, one pointset was considered as a Gaussian mixture model (GMM) centroid, the other pointset served as the data points. The GMM centroids were forced coherently as a group so that the topological structure of the pointset was preserved. By maximizing the posterior probability, the correspondence of the two pointsets was acquired and then the registration process was terminated.

Since the position and orientation of carpal bones, cartilages, and radius from one MRI scan were consistent with each other, the alignment of the radii ensured all models shared the same attitude, and hence minimizing the deviation caused by unified model posture. More rigorously, let the point set: ${𝓟}_{Rad}={\left(p_{1},\ldots ,p_{n}\right)}^{T}$ represent all the radii of all subjects acquired at all positions in RoM defined in Section 2.2. We aligned ${𝓟}_{Rad}$ to the randomly selected reference radius, ${𝓟}_{Ref}$, with the CPD algorithm. With the ensuing transformation matrix, $𝓣={\left({𝓉}_{1},\ldots ,{𝓉}_{n}\right)}^{T}$, all carpal bone model sets, ${𝓜}_{Carp}={\left(M_{1},\ldots ,M_{n}\right)}^{T}$, were aligned to the reference position with the corresponding 𝓉. Such a procedure is summarized in Algorithm 1.
**Algorithm 1:** Align the carpal bone models to the reference**Input:**${𝓟}_{Rad}$, ${𝓟}_{Ref}$, ${𝓜}_{Carp}$**Output:**𝓣, ${𝓜}_{Carp}^{\prime }$1.**Down-sample** of the ${𝓟}_{Rad}$2.Align with **CPD** such that: ${𝓟}_{Ref}\leftarrow 𝓣\cdot {𝓟}_{Rad}$3.New aligned carpal model: ${𝓜}_{Carp}^{\prime }\leftarrow 𝓣\cdot {𝓜}_{Carp}$


For the following carpal kinematic analysis, we regarded all the bones as if they were homogeneous dense solids, such that the bone volume (in mm^3^), centroid location (in mm), and the orientation of the principal inertial axes (a normalized symmetric 3 × 3 matrix) can be calculated through the models given in Section 2.3. As involved carpal bones were captured at different wrist positions, the volume of each carpal bone is given as the mean value of that bone in all positions.

**Figure 5:**
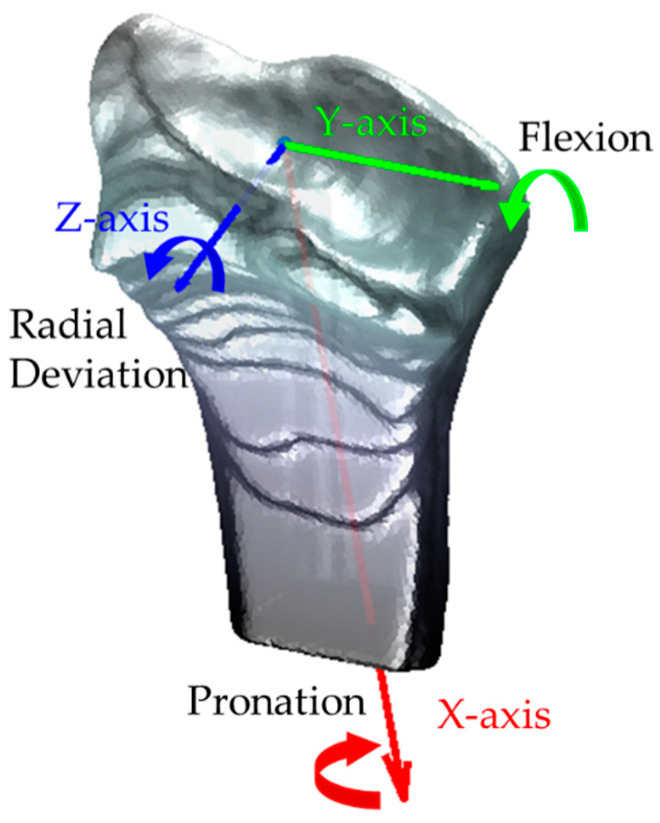
Definition of the radius-based coordinate system, positive axis direction, and wrist motions.

**Figure 6:**
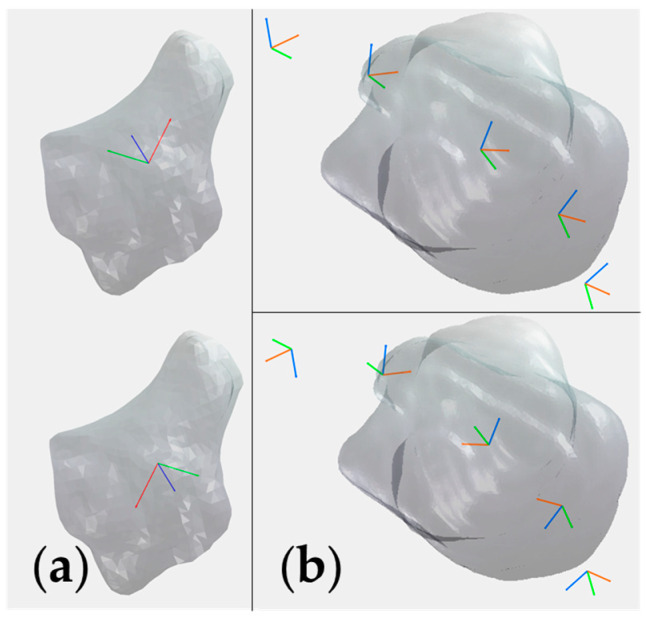
(**a**) Different coordinate system representations of the principal inertial axes for the same trapezium. (**b**) A trapezium and its five centroid positions with unified (**upper**) and ununified (**bottom**) principal inertial axes orientation in FE.

We defined the coordinate system similarly to [24]. For the radius, the *X*-axis goes through the centroid of the radius and parallel to the radial long axis, indicating pronation and supination. The *Y*-axis lies perpendicular to the *X*-axis and crosses the radial styloid, indicating the FE. The *Z*-axis is the cross product of the *X*- and *Y*-axis, and aims palmarly, indicating the RUD. The origin overlaps the intersection of the *X*-axis and the distal radial epicondyle (Figure 5). As for the coordinate system of the carpal bones, we simply utilized three principal inertial axes instead; the origin overlaps the centroid of the carpal bone. Although three principal inertial axes are unique in every bone model, the positive direction may vary individually (Figure 6a). Hence, for computational convenience, we constrained the three axes directions of one carpal bone from the same subject at different positions of one wrist positioning protocol to be the same, for instance, the direction of the coordinate system for the trapezium from subject 1 in FE stays constant (Figure 6b).

### 2.5. Motion Analysis

#### 2.5.1. Helical Axis

The HA offers a more intuitive way to illustrate the carpal bone kinematics. In this manner, the relative motion of a rigid body is decomposed into a rotation around a new axis in space and a translation along that axis. We determined the HA for the motion between the neutral position as the reference and each position during the motion.

The common method to calculate the HA and required landmarks have been explained in [25,26]. Due to the intrinsic unique characteristics, the centroid and three principal inertial axes of every carpal bone at different positions are chosen as the markers for HA calculation. The accuracy of the generated HA is highly dependent on the spatially reconstructed markers [17] and directly related to the rotation matrix [26], which can be guaranteed by our registration procedure. Thereafter, we categorized each carpal bone of the same motion (FE or RUD) from all subjects into separate groups and then applied the calculation of the HA.

We further utilized the rotation angle around and the translation along the HA for both hands to analyze the HA. As the HA is related to a rigid transformation matrix [26], we decomposed the rotation around the HA into angles around three axes, i.e., FE, RUD, and pro-/supination, which detailed the exact movement of each carpal bone during the FE and RUD.

#### 2.5.2. Carpal Bone-Radius Distance

The relative distance between bones could potentially affect carpal kinematics. For a given centroid, $C_{a}$, of one carpal bone and the origin on the radius $O_{R}$, we defined the Euclidean distance as the inter-bone distance:

(1) $$D_{inter-bone}={\left\|C_{a}-O_{R}\right\|}_{2},$$

Such descriptions can characterize the carpal bone-radius movement and, therefore, supplement the HA analysis.

#### 2.5.3. Statistical Analysis

In our study, both left and right hands of the subjects are acquired under the wrist positioning protocol. To report the difference between them, a paired-sample *t*-test is applied to characterize the difference. For the given paired *n* motion data sets: $M_{L}=\left(L_{1},\ldots ,L_{n}\right)$ for left hand and $M_{R}=\left(R_{1},\ldots ,R_{n}\right)$ for the right hand at the same wrist position, we calculate the *t* statistic as:

(2) $$t=\frac{\bar{D}}{\hat{\sigma }/\sqrt{n}},$$

where the $\bar{D}$ and $\hat{\sigma }$ are the mean difference between $M_{L}$ and $M_{R}$, and the SD of the differences, respectively. The ensuing p<0.05 is used to test for significant differences between left and right hands.

## 3. Results

**Figure 7:**
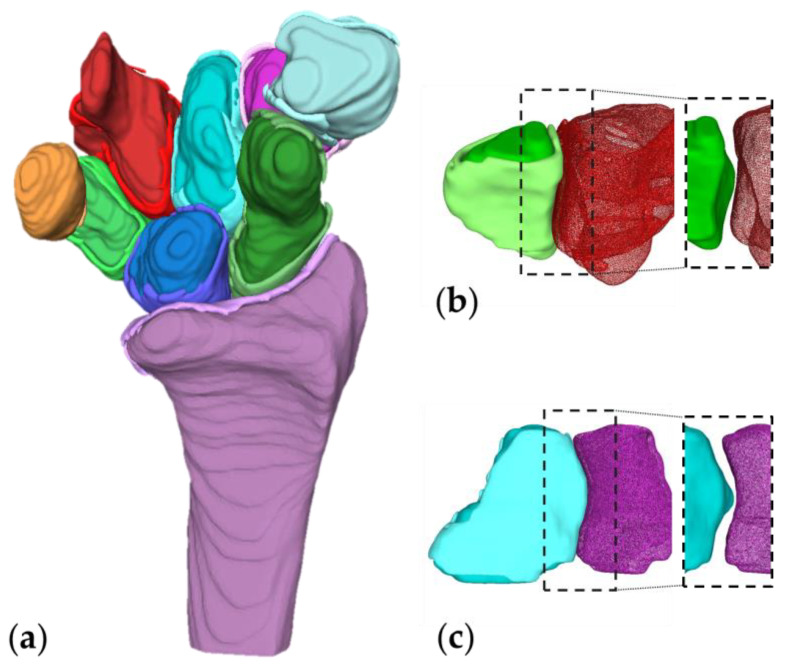
(**a**) Right carpal bones and radius with their cartilages. (**b**) A left triquetrum in green with its cartilage in light green and the adjacent hamate with its cartilage in red points. The same bones without cartilages are shown in the right dashed box. (**c**) A left capitate in cyan with its cartilage in light cyan and the adjacent trapezoid with its cartilage in magenta points. The same bones without cartilages are shown in the right dashed box.

The right carpal bones and radius with their corresponding inter-bone cartilages from one subject are shown in Figure 7a. Figure 7b,c detail the cartilages of a left trapezium and a left capitate from subject 3 and their adjacent bones. From the dashed boxes on the right side, it can be seen that the cartilages fill in the inter-bone gap.

**Figure 8:**
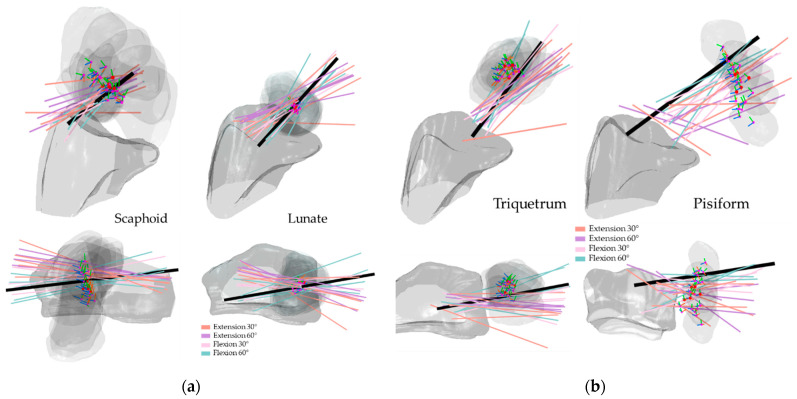
HA of the proximal carpal row in FE: (**a**) the scaphoid and lunate; (**b**) the triquetrum and pisiform. The illustrated are from a left wrist. The upper shows a side palmer view, while the bottom shows a transverse view from distal to proximal. The bold black line shows the average direction of the HAs in FE. Short RGB bars and red dots indicate the orientations and centroids of the same carpal bone from all subjects. The carpal bone from its five FE positions was randomly selected from the subjects to demonstrate the bone movement.

**Figure 9:**
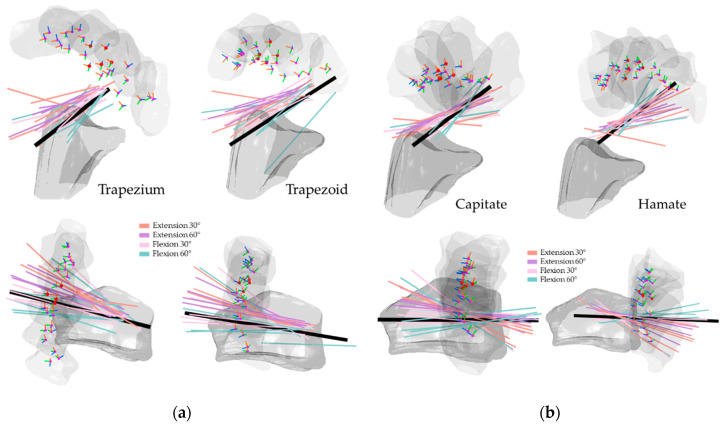
HA of the distal carpal row in FE: (**a**) the trapezium and trapezoid; (**b**) the capitate and hamate. The illustrated are from a left wrist. The upper shows a side palmer view, while the bottom shows a transverse view from distal to proximal. The bold black line shows the average direction of the HAs in FE. Short RGB bars and red dots indicate the orientations and centroids of the same carpal bone from all subjects. The carpal bone from its five FE positions was randomly selected from the subjects to demonstrate the bone movement.

**Figure 10:**
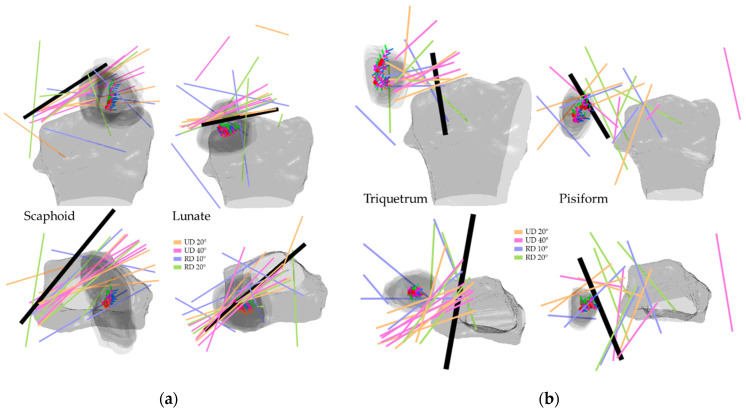
HA of the proximal carpal row in RUD: (**a**) the scaphoid and lunate; (**b**) the triquetrum and pisiform. The illustrated are from a right wrist. The upper shows a side palmer view, while the bottom shows a transverse view from distal to proximal. The bold black line shows the average direction of the HAs in RUD. Short RGB bars and red dots indicate the orientations and centroids of the same carpal bone from all subjects. The carpal bone from its five RUD positions was randomly selected from the subjects to demonstrate the bone movement.

**Figure 11:**
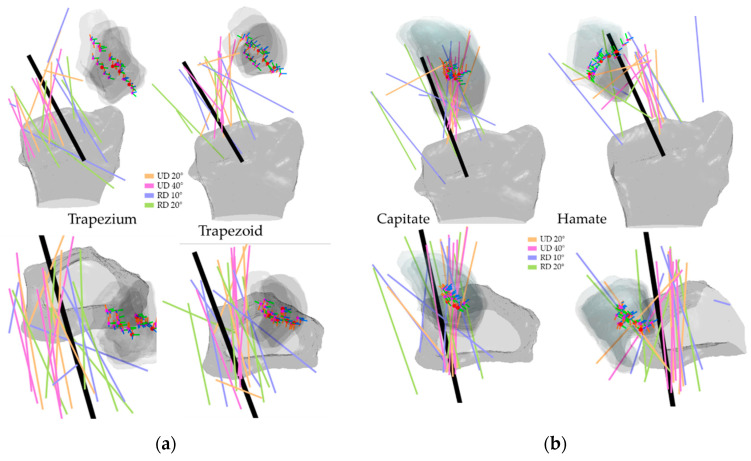
HA of the distal carpal row in RUD: (**a**) the trapezium and trapezoid; (**b**) the capitate and hamate. The illustrated are from a right wrist. The upper shows a side palmer view, while the bottom shows a transverse view from distal to proximal. The bold black line shows the average direction of the HAs in RUD. Short RGB bars and red dots indicate the orientations and centroids of the same carpal bone from all subjects. The carpal bone from its five RUD positions was randomly selected from the subjects to demonstrate the bone movement.

Figure 8, Figure 9, Figure 10 and Figure 11 illustrate the HA of the individual carpal bone of different wrist motions at different positioning angles listed in Section 2.2. Generally, during the FE, all the HAs are comparable to the *y*-axis of the radius reference and crowd as clusters. Through the transverse view, the HAs of FE intersect for all eight carpal bones, implying that the carpal bones deviate ulnarly during the flexion motion. Regarding the rotation pivot, the scaphoid, lunate, and triquetrum mainly rotate around themselves near their centroid. The rotation axes of the rest carpal bones lie relative away from their centroids, slightly above the carpal articular surface on the radius. As its centroids concentrate within a small range, the translation of the lunate relative to radius is the least among all the carpal bones, followed by the triquetrum and the scaphoid. The rest of the carpal bones show a clear arc-shaped trace corresponding to the hand movement in FE.

During the RUD, the scaphoid, lunate, and triquetrum mainly appear in a similar motion as the FE but strongly incline to the *z*-axis of the radius reference. The pisiform has a more complex rotation as the HA spread irregularly; however, the centroids stay relatively still, and the rotation is approximate more to the *z*-axis of the radius reference. The other four carpal bones in the distal row perform analogously to each other, as they deviate from radially to ulnarly. For all carpal bones, the HA of radial deviation (RD) 10° and RD 20° appear more untidily, compared to UD 20° and UD 40°. The carpal bones extend during the ulnar deviation. The lunate and pisiform have the least translation relative to the radius due to their clustered centroids. The rest carpal bones mainly translate on the coronal plane and also have an arc-shaped distribution, corresponding to the hand movement in RUD.

Table 1, Table 2, Table 3, Table 4, Table 5, Table 6, Table 7 and Table 8 list the statistical values regarding the rotation angle and translation of each carpal bone for all the wrist motions. It can be seen that carpal bones rotate around their HA together with the global wrist motions, e.g., carpal bones flex around their HAs when the hand flexes. For both hands, the capitate has the most average rotation angles among all the bones, followed by the hamate. The translations along the HAs of all the bones are typically reported within 3 mm. Among all 128 *p*-values concerning differences between the left and right hand, no significance has been noticed (all *p*-values > 0.05).

**Figure 12:**
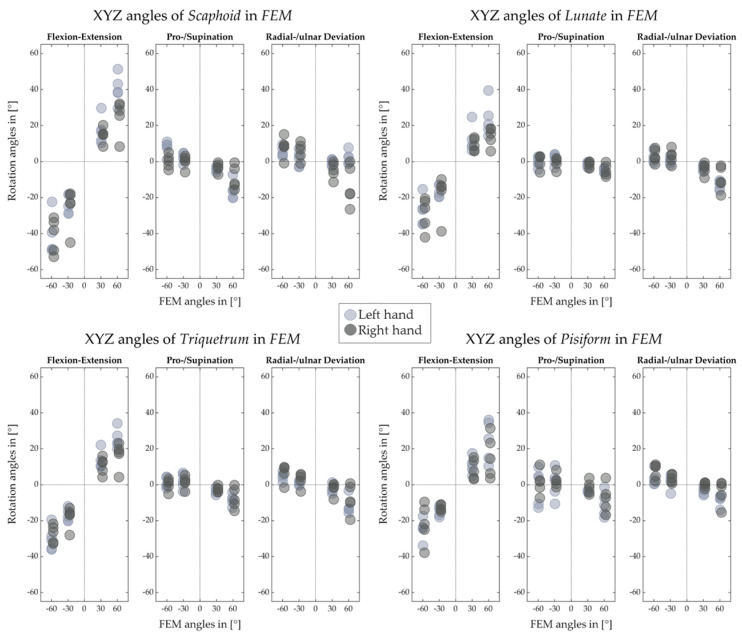
Decomposed rotation angle around the HA of proximal carpal row for left and right hand of five subjects in FE.

**Figure 13:**
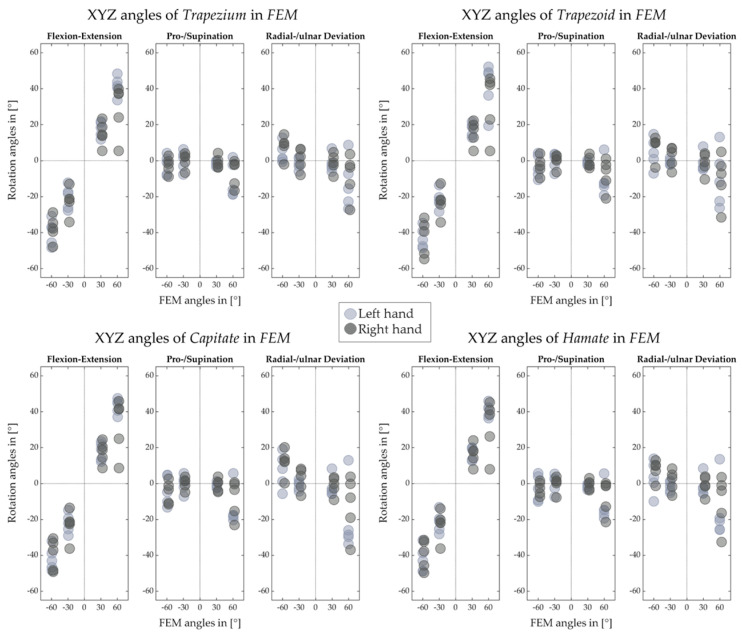
Decomposed rotation angle around the HA of distal carpal row for left and right hand of five subjects in FE.

**Figure 14:**
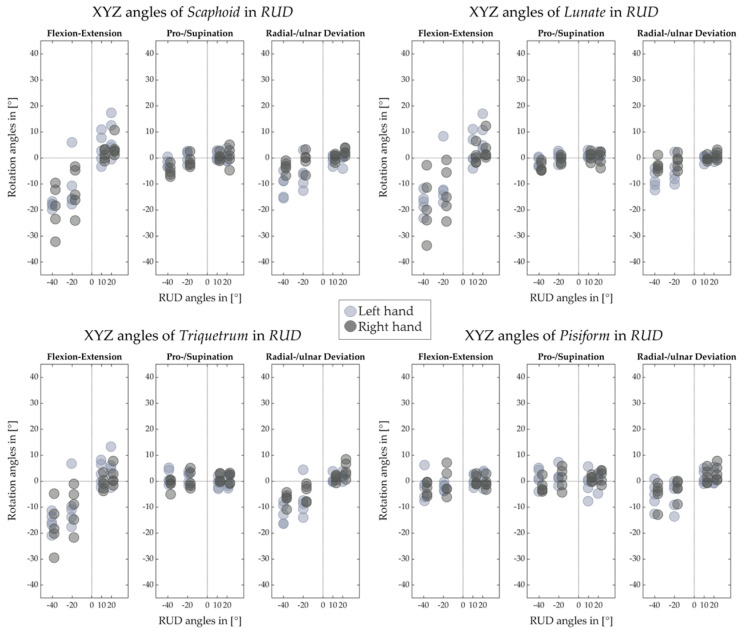
Decomposed rotation angle around the HA of proximal carpal row for left and right hand of five subjects in RUD.

**Figure 15:**
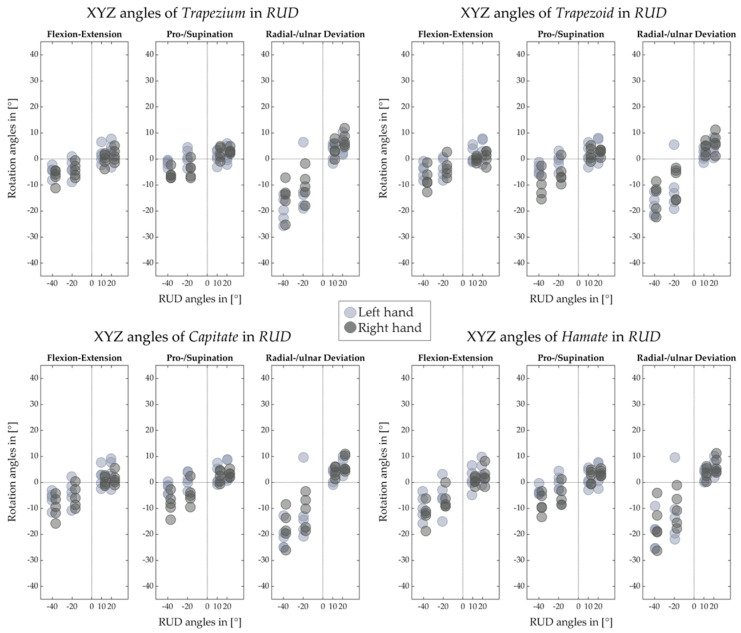
Decomposed rotation angle around the HA of distal carpal row for left and right hand of five subjects in RUD.

Figure 12, Figure 13, Figure 14 and Figure 15 illustrate the decomposed rotation angles around the HA into three rotations, which quantify the HA analysis. Figure 12 and Figure 13 confirm that during the FE, carpal bones deviate ulnarly while they extend, and per contra while flexing. The carpal bones in the proximal row perform similarly to the distal row but with smaller degrees. During the RUD, Figure 14 emphasizes the scaphoid, lunate, and triquetrum flex and extend primarily than deviate. The distal row moves likewise. All the figures demonstrate that the motion of the carpal bones during the FE and RUD is combined and multi-dimensional.

Table 9 lists the bone volumes. The volume of the same carpal bone varies differently among the subjects. For each subject, the biggest three carpal bones are the capitate, the hamate, and the scaphoid. The smallest bone is the pisiform.

**Figure 16:**
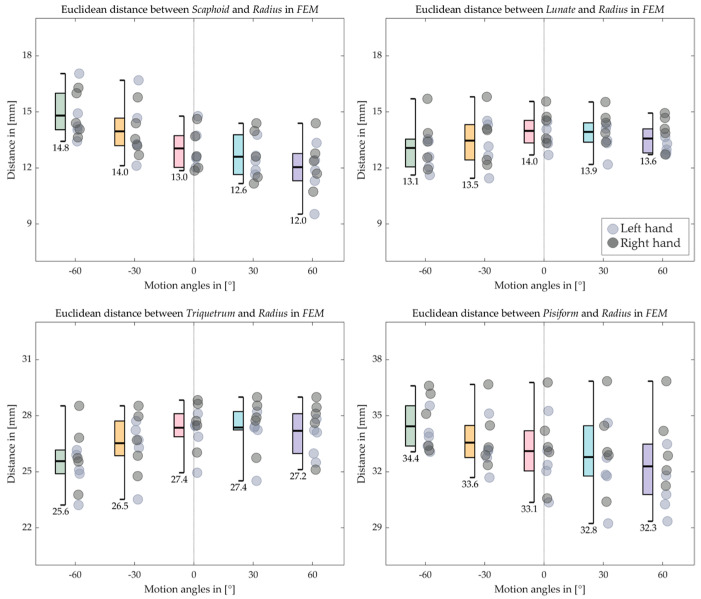
Boxplot for the Euclidean distance between the proximal carpal row and the radius in FE. The short bars outside the box indicate the maximum and the minimum distance, while the long bold bar inside draws the mean value, which is also shown under the minimum bar.

**Figure 17:**
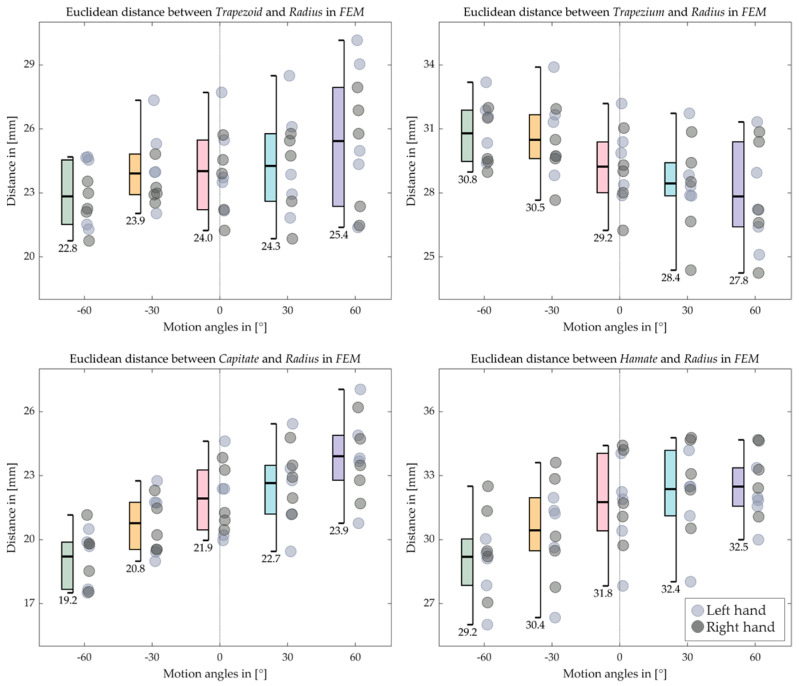
Boxplot for the Euclidean distance between the distal carpal row and the radius in FE. The short bars outside the box indicate the maximum and the minimum distance, while the long bold bar inside draws the mean value, which is also shown under the minimum bar.

**Figure 18:**
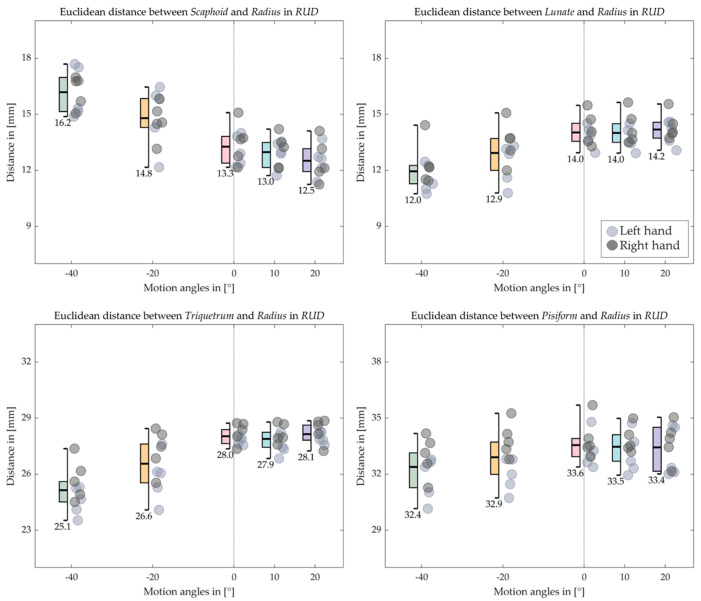
Boxplot for the Euclidean distance between the proximal carpal row and the radius in RUD. The short bars outside the box indicate the maximum and the minimum distance, while the long bold bar inside draws the mean value, which is also shown under the minimum bar.

**Figure 19:**
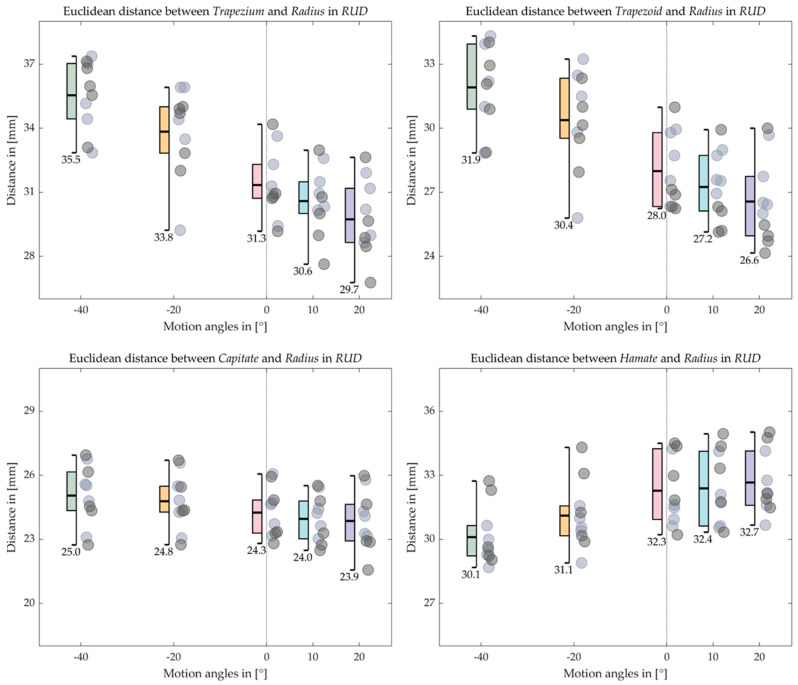
Boxplot for the Euclidean distance between the distal carpal row and the radius in RUD. The short bars outside the box indicate the maximum and the minimum distance, while the long bold bar inside draws the mean value, which is also shown under the minimum bar.

Figure 16, Figure 17, Figure 18 and Figure 19 indicate the Euclidean distance between every left and right carpal bone and the same radius reference origin of the FE and RUD. During FE, the translation of the lunate relative to the radius coheres with the analysis of the HA that the lunate moves the least among the carpal bones. The triquetrum behaves similarly to the lunate in that at the extreme wrist positions (flexion 60° and extension 60°), they move closer to radius than the intermediate positions (flexion 30° and extension 30°), respectively. The scaphoid, the pisiform, and the trapezium shift toward the radius from extension 60° to flexion 60°, while the trapezoid, the capitate, and the hamate move conversely. For the RUD, the Euclidean distance also verifies the HA analysis that the lunate and pisiform have the least translation. The scaphoid, the trapezium, and the trapezoid get closer to the radius from UD 40° to RD 20°. On the other hand, the triquetrum and hamate displace from proximal to distal.

## 4. Discussion

As Figure 7 illustrates, the inter-bone gap can be eliminated by the cartilages. Such a result is essential for further anatomical study, especially for kinematical study, as the filled inter-bone space serves as an extra boundary condition for bone motion analysis. With the additional spatial constraints, an under-defined simulation model can be optimized such that the bone element is obliged to follow a certain movement range through the motion simulation. The simulation outcome lays the foundations of a more accurate or patient-specific implant design.

Since the entire wrist motion has been *in-vivo* captured, our study offers the kinematic information with much higher accuracy and fidelity, compared with the *in-vitro* studies, as they suffered from potential disturbance caused by transducers, markers, etc., [27]. Furthermore, as we align the radii from all datasets by transforming them into point clouds (more than 15,900 points for every radius) and then align them with a more advanced algorithm, a much higher alignment accuracy can be achieved than the conventional limited anatomical landmark-based registration procedure, and hence much more precise movement can be depicted. Such advantages are essential for HA analysis due to its sensitivity to motion errors [28]. Additionally, as we create a fully automatic pipeline for the alignment, compared with traditional manual landmark detection, a bare minimum of human labor is involved in our study.

The HAs of all the carpal bones for the FE reveal certain patterns. Most of the HAs gather tightly and only a few axes locate outside the major HA cluster, however, they still aim in the same direction as the major groups, indicating that all eight carpal bones rotate simultaneously relative to the radius reference, which is also confirmed by the rotation angles listed from Table 1, Table 2, Table 3, Table 4, Table 5, Table 6, Table 7 and Table 8. Coinciding with the previous works [9,19,29], the HAs analysis of carpal bones in the proximal row confirms that during the RUD, the movement of the scaphoid, lunate, and triquetrum consists of flexion and extension motion, which implies they flex and extend regardless of the kind of wrist motion.

Figure 12, Figure 13, Figure 14 and Figure 15 for decomposed rotation angle strengthen the HA analysis. Slight bone pronation and supination during the FE and RUD have been found, which are in agreement with previous works [10,11]. Similar to previous works [9,10,11,19,27,29], complicated carpal rotations are confirmed during the wrist FE and RUD. When the wrist deviates from ulnar to radial, besides rotating ulnarly to radially, the distal carpal row also performs extension to flexion and pronation to supination. The proximal carpal row performs similarly to the distal carpal row but with less deviation and much more flexion and extension. From wrist flexion to extension, distal and proximal carpal row possesses a comparable motion. We list the limited translation of every carpal bone along their HAs in Table 1, Table 2, Table 3, Table 4, Table 5, Table 6, Table 7 and Table 8, which is agreed by previous studies [27,30] as well. The HA patterns of each carpal bone can serve as the assessment after hand surgery.

The HAs for the RUD contain a certain level of disorder. Irregular HAs are almost only found in the proximal row under the small rotation angles, i.e., RD 10° and RD 20°. The HAs of the distal row carpal bones form certain patterns, also by the RD 10° and RD 20°. Additionally, no such phenomena appear in the HAs of the FE. We, therefore, would argue that the perturbation during the image acquisition, e.g., natural upper-limb movement of the subject during the acquisition, causes such a HA distribution.

We further apply statistical analysis to the kinematic difference between the left and right hand. For two parameters characterizing the motion of the HA, we calculated the *p*-value of every carpal bone of both hands. Among 128 *p*-values, no significance has been found, statistically confirming the hypothesis that there is no difference in motion pattern between left and right hands.

Table 9 reports the volume of different carpal bones, which corresponds to the previous studies [8,18,30]. We observe the volumes of the scaphoid, lunate, and triquetrum of the involved female (Sub 5) are slightly smaller than the males (Sub 1–4); however, it does not show any difference between other bones. In Figure 10, Figure 11 and Figure 14, we also notice the bone centroid distribution of subject 5 has a relatively shorter distance toward radius reference, which implies a relatively smaller carpal volume. The reason has been reported in [8] why the bone volume of females could be smaller on average than males but with exceptions.

We quantified inter-bone space characteristics with carpal bone-radius distance, which strengthened our HA analysis regarding bone movement. For both FE and RUD, carpal bones in the distal row make greater movement than in the proximal row, which is rational, since the rotation centers are roughly located near the radius. Despite relatively small displacement, carpal bone in the proximal row translates longer in RUD than in FE, as the mean distance between lunate and radius increases on average from within 0.5 mm in FE to within 2.2 mm in RUD. The same trend also appears in the distal row as the trapezoid-to-radius distance increases from within 3 mm in FE to within 5.5 mm in RUD. Taking the lunate for instance, as shown in Figure 8 and Figure 10, the HAs in FE concentrate inside the lunate near its centroid, while the HAs in RUD are found above the lunate surface; such HA distributions imply the lunate pivot is closer to its centroid in FE, and thus have less translation.

As confirmed by the studies from [1,2,8,9,10,11], carpal kinematics can be studied statically in individuals without wrist injuries. Our study covers the normal daily RoM of the human wrist and, as illustrated in Section 3 and above, we can analyze the carpal kinematics with the acquired MR images. However, due to currently limited subjects, despite the legible motion pattern in FE, inferior HA results are noticed in the pisiform in RUD. Additionally, our current model includes wrists from limited age groups. Studies on wrist models from young and aged subjects are still expected. Including more subjects in the following studies is, therefore, intended. With the cartilage, we have narrowed the inter-bone gap, as well as the fidelity of the computational simulation. Hence, the introduction of the ligament, tendons, and capsular fibers remains a research topic for our further studies.

## 5. Conclusions

In this paper, we studied the kinematics of all carpal bones based on MR images. Our proposed wrist models possess the cartilage information for the first time. The study visualizes the HA *in-vivo* for all eight carpal bones for the first time. The further detailed analysis regarding rotation angle and translation confirms the motion pattern of the commonly studied carpal bones, e.g., scaphoid, lunate, and capitate, and extends investigations to all other carpal bone motion during the FE and RUD, revealing a more comprehensive understanding of carpal kinematics. We further statistically confirm the hypothesis that there is no difference in motion pattern between left and right hands.

With the knowledge revealed in the proposed study, we believe a better understanding of carpal motion based on a healthy wrist can be obtained for further pathologic assessment and surgical treatment.

## Figures and Tables

**Table 1:** Rotation angle around and translation along the HA of scaphoid for both hands, left hand, and right hand of five subjects. The *p*-value is given between the left and right hands. The R, T, avg, and sd represent rotation angle in degree [°], translation in [mm], mean value, and standard deviation, respectively.

		Scaphoid															
		E60°		E30°		F30°		F60°		UD40°		UD20°		RD10°		RD20°	
		R	T	R	T	R	T	R	T	R	T	R	T	R	T	R	T
Both	avg	−42.20	−1.35	−25.39	−0.79	16.97	−0.17	36.30	−0.25	−14.42	−0.87	−20.98	−1.78	4.06	−0.09	7.11	−0.05
	sd	10.19	0.58	8.35	0.61	6.07	0.26	12.21	0.39	6.74	0.71	6.24	0.58	3.41	0.35	5.45	0.61
Left	avg	−47.38	−1.49	−25.69	−0.51	14.90	−0.14	41.18	0.00	−14.74	−0.96	−22.24	−1.56	5.86	0.00	9.10	−0.18
	sd	4.44	0.67	4.95	0.80	3.25	0.26	5.56	0.23	5.74	1.05	1.47	0.62	5.10	0.16	7.68	0.22
Right	avg	−38.76	−1.25	−25.19	−0.98	18.35	−0.19	33.05	−0.42	−14.21	−0.81	−20.14	−1.93	2.86	−0.15	5.77	0.03
	sd	11.81	0.56	10.51	0.42	7.37	0.28	14.77	0.39	7.87	0.48	8.17	0.56	1.00	0.44	3.57	0.79
*p*-value		0.96	0.81	0.75	0.45	0.68	0.21	0.13	0.85	0.64	0.57	0.74	0.14	0.22	0.66	0.55	0.48

**Table 2:** Rotation angle around and translation along the HA of lunate for both hands, left hand, and right hand of five subjects. The *p*-value is given between the left and right hands. The R, T, avg, and sd represent rotation angle in degree [°], translation in [mm], mean value, and standard deviation, respectively.

		Lunate															
		E60°		E30°		F30°		F60°		UD40°		UD20°		RD10°		RD20°	
		R	T	R	T	R	T	R	T	R	T	R	T	R	T	R	T
Both	avg	−28.57	−1.12	−17.93	−0.56	11.67	0.02	22.49	0.36	−13.92	−0.81	−19.41	−1.72	4.08	−0.07	6.57	−0.27
	sd	8.15	0.51	8.15	0.63	5.64	0.20	8.94	0.63	6.94	0.78	8.22	0.77	3.49	0.40	5.16	0.59
Left	avg	−30.86	−1.13	−17.74	−0.21	9.76	0.05	23.91	0.79	−14.82	−1.10	−21.28	−1.76	5.19	0.03	8.75	−0.11
	sd	4.87	0.50	2.97	0.67	2.55	0.13	4.36	0.43	4.81	1.02	2.29	0.69	5.25	0.17	6.77	0.19
Right	avg	−27.05	−1.12	−18.05	−0.80	12.95	−0.01	21.55	0.07	−13.33	−0.61	−18.17	−1.69	3.34	−0.14	5.11	−0.38
	sd	9.91	0.56	10.69	0.54	6.97	0.25	11.39	0.59	8.46	0.59	10.67	0.89	1.92	0.51	3.74	0.75
*p*-value		0.82	0.8	0.76	0.57	0.64	0.2	0.18	0.55	0.8	0.35	0.94	0.93	0.52	0.69	0.6	0.43

**Table 3:** Rotation angle around and translation along the HA of triquetrum for both hands, left hand, and right hand of five subjects. The *p*-value is given between the left and right hands. The R, T, avg, and sd represent rotation angle in degree [°], translation in [mm], mean value, and standard deviation, respectively.

		Triquetrum															
		E60°		E30°		F30°		F60°		UD40°		UD20°		RD10°		RD20°	
		R	T	R	T	R	T	R	T	R	T	R	T	R	T	R	T
Both	avg	−29.56	−1.18	−18.18	−0.53	12.86	0.21	24.56	0.77	−13.39	−0.26	−19.52	−1.26	4.04	0.16	6.46	0.09
	sd	5.77	0.78	4.52	0.58	4.90	0.27	8.47	0.58	6.79	0.76	6.71	0.64	2.61	0.48	3.57	0.46
Left	avg	−33.29	−1.22	−18.18	−0.20	12.28	0.12	27.97	1.05	−15.45	−0.59	−22.05	−1.28	5.05	0.09	7.51	0.06
	sd	3.51	0.87	3.95	0.69	1.53	0.24	1.71	0.57	5.87	0.84	0.88	0.19	3.91	0.47	4.21	0.31
Right	avg	−27.07	−1.16	−18.18	−0.74	13.25	0.27	22.28	0.59	−12.01	−0.03	−17.83	−1.24	3.36	0.20	5.75	0.12
	sd	5.83	0.81	5.23	0.43	6.44	0.30	10.58	0.55	7.53	0.67	8.48	0.85	1.32	0.52	3.29	0.57
*p*-value		0.62	0.74	0.73	0.54	0.5	0.59	0.14	0.64	0.49	0.59	0.62	0.66	0.52	0.07	0.71	0.98

**Table 4:** Rotation angle around and translation along the HA of pisiform for both hands, left hand, and right hand of five subjects. The *p*-value is given between the left and right hands. The R, T, avg, and sd represent rotation angle in degree [°], translation in [mm], mean value, and standard deviation, respectively.

		Pisiform															
		E60°		E30°		F30°		F60°		UD40°		UD20°		RD10°		RD20°	
		R	T	R	T	R	T	R	T	R	T	R	T	R	T	R	T
Both	avg	−25.56	0.65	−15.39	0.67	10.68	0.34	23.77	1.31	−7.07	−0.74	−7.96	−0.56	3.93	−0.09	4.77	0.15
	sd	7.98	3.17	2.69	1.69	4.87	0.91	11.62	1.50	4.19	1.60	4.04	0.67	3.01	0.71	2.51	1.12
Left	avg	−27.95	−0.29	−16.39	0.42	12.39	0.61	31.13	1.40	−8.08	−0.60	−9.30	−0.83	3.44	−0.14	3.77	0.15
	sd	4.42	3.24	2.92	1.11	4.72	0.66	9.91	1.30	5.60	1.86	4.20	0.75	3.31	0.62	2.41	0.86
Right	avg	−23.97	1.28	−14.73	0.85	9.53	0.16	18.86	1.24	−6.40	−0.83	−7.06	−0.37	4.26	−0.05	5.44	0.15
	sd	9.77	3.25	2.57	2.08	5.04	1.06	10.58	1.73	3.38	1.58	4.05	0.61	3.07	0.81	2.56	1.34
*p*-value		0.65	0.22	0.22	0.71	0.25	0.08	0.06	0.28	0.9	0.71	0.3	0.73	0.59	0.13	0.75	0.32

**Table 5:** Rotation angle around and translation along the HA of trapezium for both hands, left hand, and right hand of five subjects. The *p*-value is given between the left and right hands. The R, T, avg, and sd represent rotation angle in degree [°], translation in [mm], mean value, and standard deviation, respectively.

		Trapezium															
		E60°		E30°		F30°		F60°		UD40°		UD20°		RD10°		RD20°	
		R	T	R	T	R	T	R	T	R	T	R	T	R	T	R	T
Both	avg	−39.56	−1.58	−22.33	−1.41	17.07	−0.44	37.97	−0.95	−13.69	−1.45	−19.17	−2.19	5.16	−0.52	7.92	−0.31
	sd	6.83	1.04	6.49	1.04	5.61	0.73	13.87	1.92	5.47	0.91	5.43	0.95	3.01	0.55	3.60	0.95
Left	avg	−42.43	−1.72	−21.36	−0.95	17.83	−0.34	47.34	−1.09	−14.57	−1.33	−21.47	−1.88	5.11	−0.72	8.08	−0.87
	sd	6.17	0.66	7.36	0.59	5.15	0.49	5.30	3.02	5.60	0.91	4.00	0.49	3.58	0.69	4.71	0.99
Right	avg	−37.64	−1.50	−22.98	−1.71	16.56	−0.51	31.73	−0.86	−13.11	−1.53	−17.64	−2.40	5.20	−0.39	7.82	0.07
	sd	7.08	1.29	6.49	1.21	6.32	0.90	14.57	1.06	5.84	0.99	6.04	1.17	2.94	0.46	3.17	0.79
*p*-value		0.78	0.8	0.53	0.37	0.58	0.87	0.24	0.59	0.52	0.56	0.44	0.4	0.4	0.17	0.6	0.25

**Table 6:** Rotation angle around and translation along the HA of trapezoid for both hands, left hand, and right hand of five subjects. The *p*-value is given between the left and right hands. The R, T, avg, and sd represent rotation angle in degree [°], translation in [mm], mean value, and standard deviation, respectively.

		Trapezoid															
		E60°		E30°		F30°		F60°		UD40°		UD20°		RD10°		RD20°	
		R	T	R	T	R	T	R	T	R	T	R	T	R	T	R	T
Both	avg	−43.62	−1.95	−23.04	−1.22	17.34	−0.45	39.45	−1.52	−13.24	−1.78	−19.75	−2.90	4.67	−0.34	7.55	−0.46
	sd	7.77	0.53	6.16	0.97	5.34	0.67	16.47	1.53	5.58	0.92	5.41	1.08	3.16	0.91	3.95	1.02
Left	avg	−45.54	−2.08	−21.66	−0.82	17.51	−0.32	45.86	−1.86	−14.44	−1.67	−20.28	−2.60	4.48	−0.98	8.20	−0.86
	sd	4.61	0.41	6.07	0.76	4.25	0.25	15.29	1.92	5.80	0.83	1.92	0.50	4.17	0.86	5.66	1.10
Right	avg	−42.35	−1.87	−23.95	−1.48	17.23	−0.53	35.18	−1.28	−12.44	−1.85	−19.40	−3.11	4.79	0.09	7.12	−0.20
	sd	9.54	0.62	6.61	1.06	6.36	0.87	17.12	1.35	5.84	1.04	7.08	1.35	2.75	0.71	2.88	0.97
*p*-value		0.99	0.83	0.47	0.44	0.72	0.78	0.51	0.17	0.62	0.87	0.84	0.35	0.64	0.07	0.92	0.62

**Table 7:** Rotation angle around and translation along the HA of capitate for both hands, left hand, and right hand of five subjects. The *p*-value is given between the left and right hands. The R, T, avg, and sd represent rotation angle in degree [°], translation in [mm], mean value, and standard deviation, respectively.

		Capitate															
		E60°		E30°		F30°		F60°		UD40°		UD20°		RD10°		RD20°	
		R	T	R	T	R	T	R	T	R	T	R	T	R	T	R	T
Both	avg	−42.30	−1.25	−23.38	−1.15	18.38	−0.44	42.73	−0.27	−15.45	−1.58	−22.29	−2.64	5.32	−0.28	8.81	−0.32
	sd	7.60	0.79	6.41	0.92	5.58	0.46	14.93	1.37	5.67	0.90	6.12	0.92	3.35	0.60	4.18	0.92
Left	avg	−45.32	−1.61	−22.44	−0.87	18.33	−0.03	52.13	0.86	−16.39	−1.59	−23.59	−2.44	6.25	−0.52	10.29	−0.76
	sd	5.01	0.50	6.41	0.70	5.75	0.32	2.83	0.85	4.65	0.98	2.11	0.63	4.57	0.60	5.52	0.84
Right	avg	−40.29	−1.01	−24.00	−1.33	18.41	−0.71	36.47	−1.02	−14.81	−1.56	−21.42	−2.78	4.70	−0.11	7.82	−0.02
	sd	8.76	0.89	6.94	1.06	6.02	0.32	16.70	1.12	6.60	0.93	7.91	1.11	2.55	0.59	3.21	0.93
*p*-value		0.77	0.77	0.68	0.5	0.82	0.26	0.26	0.34	0.61	0.99	0.98	0.4	0.61	0.07	0.72	0.26

**Table 8:** Rotation angle around and translation along the HA of hamate for both hands, left hand, and right hand of five subjects. The *p*-value is given between the left and right hands. The R, T, avg, and sd represent rotation angle in degree [°], translation in [mm], mean value, and standard deviation, respectively.

		Hamate															
		E60°		E30°		F30°		F60°		UD40°		UD20°		RD10°		RD20°	
		R	T	R	T	R	T	R	T	R	T	R	T	R	T	R	T
Both	avg	−41.71	−1.79	−22.99	−1.28	17.43	−0.49	40.19	−0.34	−15.94	−1.31	−23.01	−2.36	5.16	−0.29	8.49	−0.14
	sd	7.35	0.75	6.64	0.90	5.12	0.50	13.28	1.40	5.90	1.07	6.40	1.20	3.01	0.55	4.31	1.15
Left	avg	−45.51	−2.17	−22.01	−0.97	17.01	−0.08	47.28	0.71	−17.27	−1.42	−24.10	−2.10	5.42	−0.65	9.02	−0.59
	sd	5.22	0.56	6.65	0.66	4.32	0.28	2.17	0.96	5.20	1.30	2.34	0.58	4.07	0.56	5.99	1.01
Right	avg	−39.17	−1.53	−23.64	−1.48	17.71	−0.76	35.47	−1.04	−15.05	−1.24	−22.29	−2.52	4.99	−0.06	8.13	0.17
	sd	7.84	0.80	7.17	1.04	5.98	0.43	15.74	1.22	6.64	1.01	8.29	1.52	2.50	0.43	3.39	1.23
*p*-value		0.63	0.78	0.66	0.58	0.83	0.21	0.27	0.38	0.5	0.73	0.98	0.11	0.46	0.06	0.98	0.19

**Table 9:** Bone volumes in mm^3^ of involved subjects.

	Scaphoid	Lunate	Triquetrum	Pisiform	Trapezium	Trapezoid	Capitate	Hamate
Sub 1	2309.7	1699.8	1363.2	622.4	1673.0	1095.9	3243.0	2141.0
Sub 2	2411.4	1569.1	1389.9	996.7	2012.0	1345.8	3389.4	2890.7
Sub 3	2216.7	1632.1	1245.4	640.9	1865.8	1152.5	3249.2	2529.6
Sub 4	2073.8	1733.2	1258.6	631.1	1494.9	1233.4	2862.5	2446.8
Sub 5	1849.2	1184.0	1277.0	637.3	1695.8	1152.1	2643.1	2443.4
mean	1961.5	1458.6	1267.8	634.2	1595.3	1192.7	2752.8	2445.1
SD	196.0	198.1	58.4	145.6	176.6	86.8	279.0	240.0

## Data Availability

Not applicable.
